# Cases of Cynanche Trachealis, &c.

**Published:** 1811-12

**Authors:** 


*To the Editors of the Medical and Physical Journal.*

Gentlemen,

*To the Editors of the Medical and Physical Journal.*

Gentlemen,

The following remarks occurred in perusing the last Number of the Medical and Physical Journal: should they be thought thought deserving of a corner in the next Number, they are very much at your service.

ANNOTATOR.

*Nov* 4, 1811.

P. 377.—Dr. Beaver thinks that many cases of Cynanche Trachealis in adult subjects have not been recorded: he instances only that of the late General Washington. In addition may be mentioned the cases of two eminent medical characters, the late Dr. Pitcairn and Sir John Hayes.

P. 379.—The case of Small-Pox, received by casual infection eight or nine years after the same disease had been communicated by inoculation, would have been more perfect had Dr. Storer stated from whom the matter for inoculation had been taken:–whether from the natural or the inoculated small-pox; and at what period of the disease. Those who deny the possibility of the small-pox being communicated twice to the same individual will, perhaps, with their wonted arrogance, assert that the boy was mistakenly inoculated with the virus of Varicella.

P. 386.—Many of your readers will, I presume, think that the physician was not very unreasonable, who requested that "the stomach might be suffered to rest," after the patient had been drenched with a draught "*every two hours*." The apothecary seems to have thought that there was no safety but in medicine: the physician supposed, (no very absurd supposition), that nourishment might likewise be of service; and we are not told that the taking of food, or the delay in giving medicines, was eventually injurious to the patient.

If apothecaries will continue, as many do, to load their patients with medicines, with unbounded profusion, they must not wonder that the patients prefer employing a physician, and send his prescriptions to a druggist; and if they will, as many do, interfere with the physician's plans, by altering his medicines, or giving others in addition to those he orders; they must not be surprised that the physician should direct his prescriptions to be taken to a druggist in future.

At the same time that I blame the apothecaries for what is not only unwarrantable, but very impolitic conduct, in overloading their patients with medicines, without considering any thing but filling their own pockets, I must acknowledge that the conduct of the physician is sometimes as blameable, in not sufficiently consulting the interests of the honest apothecary, whose services are called for to visit the patient frequently. This reminds me of a story respecting the late Dr. Fothergill, which I have been assured is a fact.

 As

As Dr. Fothergill was one morning walking to see some of his patients, he met an apothecary of his acquaintance, when a dialogue to the following purport took place between them.

*Ap.* Good morning, Doctor; I am thinking of coming to dine with you to-day.

*Dr. F.* Well, friend, I shall be glad to see thee.

*Ap.* And my wife will come with me, Doctor.

*Dr. F.* Well, friend, I do not often give dinners; but if thy wife will be content with my plain living, I shall be very glad to see her too.

*Ap.* And I must bring my children, and my shopman, and my servants with me, Doctor.

*Dr. F.* Friend, I perceive there is something about this that I do not understand. What is thy meaning?

*Ap.* Why, Doctor, you have been called in to visit my old patient, Mr. B——, who is always ailing, but never ill. Now, he *will* have me call upon him twice, and sometimes three times a day; and therefore I am obliged, in order to pay myself for my trouble, to send him three or four draughts a day, and an occasional mixture, or something of that kind. Now, Doctor, you have ordered for him a box of twenty-four pills, of which he is to take one three times a day. The pills, therefore, will last him just eight days. I reckon that the pills, the box, my shopman's time, paper, booking, &c. will cost me one shilling; so that if I charge him three shillings for the box of pills, which is as much as I can do, and make him only two visits a day during the time he is taking them, I shall receive only two shillings for sixteen visits; while you, for only one visit, receive a guinea. So you see, Doctor, that I and my family must dine at your expence, at least once a week.

*Dr. F.* Say no more, friend; thy complaint is reasonable. I will do thee more justice when I see thy patient next time.

The story says, that the good doctor kept his word, and that the apothecary was satisfied; but I cannot believe that the poor patient was drenched with a draught *every two hours*.

